# Antibiotic prescription consistent with guidelines in emergency department is associated with 30-day survival in severe community-acquired pneumonia

**DOI:** 10.1186/s12873-021-00505-4

**Published:** 2021-09-27

**Authors:** Seung Hyun Kang, You Hwan Jo, Jae Hyuk Lee, Dong-Hyun Jang, Yu Jin Kim, Inwon Park

**Affiliations:** 1 Department of Emergency Medicine, Seoul National University Bundang Hospital, 82, Gumi-ro 173 Beon-gil, Bundang-gu, Seongnam-si, Gyeonggi-do 13620 Republic of Korea; 2 Department of Emergency Medicine, Seoul National University College of Medicine, 103 Daehak-ro, Jongno-gu, Seoul, 03080 Republic of Korea; 3 Department of Emergency Medicine, Korea University Anam Hospital, 73, Goryeodae-ro, Seongbuk-gu, Seoul, 02841 Republic of Korea

**Keywords:** Community-acquired pneumonia, Emergency department, Survival, Treatment guidelines

## Abstract

**Background:**

The selection of initial empirical antibiotics is an important issue in the treatment of severe community-acquired pneumonia (CAP). This study aimed to investigate whether empirical antibiotic prescription concordant with guidelines in the emergency department (ED) affects 30-day mortality in patients with severe CAP.

**Methods:**

We conducted a retrospective analysis of adult patients with severe CAP who were hospitalized in the ED. Severe CAP was defined according to the criteria of the 2007 Infectious Diseases Society of America/American Thoracic Society guidelines. Patients were divided into two groups according to whether they were prescribed empirical antibiotics concordant with guidelines. Multivariable Cox proportional hazard regression analysis was performed to identify the independent association between the prescription of initial empirical antibiotics concordant with the guidelines and 30-day mortality. Propensity score matching was performed to reduce selection bias between groups and Kaplan–Meier survival analysis was performed to analyze the time-to-event of 30-day survival.

**Results:**

In total, 630 patients were hospitalized in the ED for severe CAP, and 179 (28.4%) died within 30 days. Antibiotics consistent with guidelines were prescribed to 359 (57.0%) patients. The 30-day mortality was significantly higher in the guideline-discordant group (*p* = 0.003) and multivariable Cox proportional hazard regression analysis revealed that the prescription of antibiotics discordant with the guidelines was independently associated with 30-day mortality (hazard ratio 1.43, 95% CI 1.05–1.93). After propensity score matching, there were 255 patients in each group. The 30-day mortality was lower in the group prescribed guideline-concordant antibiotics than in the group prescribed guideline-discordant antibiotics (23.9% vs. 33.3%, *p* = 0.024). Kaplan–Meier survival analysis showed that antibiotic prescription concordant with the guidelines resulted in higher survival rates at 30 days (*p* = 0.002).

**Conclusions:**

The prevalence of antibiotic prescription consistent with guidelines for severe CAP seemed to be low in the ED, and this variable was independently associated with 30-day survival.

## Background

Community-acquired pneumonia (CAP) is a common and potentially lethal infectious disease worldwide [1–3]. Although many studies have been conducted on CAP treatment and many treatment guidelines have been established, CAP remains a major health concern worldwide with substantial rates of morbidity and mortality [4, 5].

CAP is induced by various etiologic agents such as bacteria, fungi, viruses, and protozoa, and treatment for CAP begins with the determination of the causative pathogens. However, confirmatory microbiological test results are not immediately available and the yield of identifying the causative pathogens through sputum or blood culture is low [6, 7]. In addition, most CAPs are induced by several common causative pathogens [8]. For this reason, initial antibiotics in CAP patients are usually empirically chosen by targeting common causative pathogens [5].

Because of the diversity of causative pathogens and differences in regional antibiotic susceptibility patterns, suggested treatments may vary regionally, and several international guidelines have been published accordingly to assist physicians in determining the initial empirical antibiotics to be prescribed [5, 9, 10]. Most of these guidelines recommend empirical antibiotics based on the judgment of the severity of the patient, the need for treatment provision on an outpatient or inpatient basis, and the risk of infection with multidrug resistant organisms such as methicillin-resistant *Staphylococcus aureus* (MRSA) and *Pseudomonas aeruginosa*. Several studies have shown that the selection of empirical antibiotics in accordance with the guidelines improves outcomes such as patient survival and hospital length of stay [11–14].

Severe CAP requires admission to the intensive care unit (ICU) and close monitoring of the patient owing to the high severity and mortality [15]. For severe CAP, as with other severe infectious diseases, immediate treatments including the administration of empirical antibiotics, respiratory support, and hemodynamic resuscitation are important to reduce mortality [12, 16]. Of these approaches, the selection of empirical antibiotics based on surveillance data on the etiologies and susceptibility patterns of the most common pathogens may be one of the most important treatment measures. Most international guidelines for the treatment of CAP recommend the administration of a combination of antibiotics, including β-lactam plus macrolides or fluoroquinolones, to obtain sufficient antimicrobial coverage [5, 9, 10].

For the majority of critically ill patients, including those with severe CAP, the emergency department (ED) is an important place wherein most of the initial resuscitation procedures and treatments are performed. We hypothesized that the selection of initial empirical antibiotics according to the guidelines for patients with severe CAP in the ED would be associated with patient prognosis. Therefore, we conducted this study to investigate whether adherence to the Infectious Diseases Society of America/American Thoracic Society (IDSA-ATS) guidelines for CAP in selecting empirical antibiotics in the ED affects 30-day survival in patients with severe CAP.

## Methods

### Setting and study population

This study was a retrospective analysis of adult patients with severe CAP who were hospitalized in the ED of an urban tertiary teaching hospital between September 2013 and August 2019. The inclusion criteria were adult (18 years old or older) patients who were hospitalized after the diagnosis of and treatment for CAP in the ED and met the criteria for severe CAP. Severe CAP was defined according to the criteria of the 2007 IDSA/ATS guidelines [17]. Patients who met at least one major criterion (either septic shock or respiratory failure requiring mechanical ventilation) or three or more minor criteria (respiratory rate, ≥30 breaths/min; arterial oxygen pressure/fraction of inspired oxygen [PaO_2_/FiO_2_] ratio, ≤250; multilobar infiltrates on chest X-ray or computed tomography scan; confusion or changed mental status; blood urea nitrogen level, > 20 mg/dL; leukopenia due to infection; thrombocytopenia; hypothermia; or hypotension requiring aggressive fluid resuscitation) were included.

Patients younger than 18 years of age or those who did not meet the criteria for severe CAP were excluded. Patients transferred directly from the ED to other hospitals, patients who received immunosuppressive treatment (steroids, immunosuppressive agents, or chemotherapy) within 1 month of admission, and patients who were transferred from other hospitals for pneumonia and had already received antibiotics before visiting the ED were also excluded.

The hospital has an electronic medical record and prescription system, among which antibiotics can be selected from the recommended list according to the diagnosis of infectious diseases, or antibiotics can be searched and prescribed if appropriate antibiotics are not included on the list. For CAP, these recommendations were made according to IDSA/ATS guidelines. During the period of this study, the IDSA/ATS guidelines had not been updated since 2007. Thus, the prescription of initial empirical antibiotics was judged according to the 2007 IDSA/ATS guidelines on the management of CAP in adult patients [17]. In the guidelines, for patients with severe CAP, β-lactams (cefotaxime, ceftriaxone, or ampicillin-sulbactam) plus either a macrolide or fluoroquinolone were recommended, and for patients who were allergic to penicillin, a respiratory fluoroquinolone plus aztreonam was recommended. In addition, the guidelines recommend the use of an antipseudomonal β-lactam along with azithromycin or fluoroquinolone with or without aminoglycoside due to concerns of *P. aeruginosa* infection, and vancomycin or linezolid is added due to concerns of MRSA infection. Thus, patients who received (non-antipseudomonal or antipseudomonal) β-lactam plus macrolide, β-lactam plus fluoroquinolone or fluoroquinolone plus aztreonam when the patients were allergic to penicillin were judged as concordant with guidelines, and other prescriptions such as β-lactam without macrolide or fluoroquinolone, fluoroquinolone without β-lactam, β-lactam plus anti-anaerobes, or carbapenem only, were judged as discordant with the guidelines.

The institutional review board of our institution approved this retrospective analysis and waived the need for informed consent (B-2101/663–108).

### Data collection

Data were obtained from hospital medical records with standardized data collection forms. The data collection forms included patients’ age; sex; comorbidities such as diabetes, hypertension, chronic obstructive lung disease, congestive heart failure, chronic kidney disease, chronic liver disease, neoplastic disease, and cerebrovascular disease; hemodynamic variables such as blood pressure, heart rate, respiratory rate, and body temperature; laboratory data such as complete blood count, and serum chemistry; organisms isolated from sputum and blood cultures; and initial antibiotics prescribed in the ED. It was also determined whether the patient had accompanying septic shock requiring vasopressor administration or respiratory failure requiring mechanical ventilation at the time of ED admission.

From the collected data, the pneumonia severity index (PSI) score was calculated. Data on the initial empirical antibiotics administered in the ED were collected from medical records and judged as concordant or discordant with the IDSA/ATS CAP guidelines. Data on survival at 30 days and survival duration were gathered from medical records either during hospitalization or during outpatient follow-up after discharge. The survival duration of patients who could not be confirmed through the medical record was obtained through telephone contact with the patients or surrogates.

### Statistical analyses

The patients were divided into two groups according to whether they were prescribed empirical antibiotics concordant with the guidelines. Characteristics of the study subjects were summarized using the mean and standard deviation for continuous variables, and frequency and percentage for categorical variables. Multivariable Cox proportional hazard regression analysis was performed to identify the independent association between the prescription of initial empirical antibiotics concordant with the guidelines and 30-day mortality. Multivariable analysis included age, sex, and variables that differed between groups in univariable analysis.

To evaluate the effect of the prescription of guideline-concordant antibiotics by reducing selection bias, propensity score matching was used. Multivariable logistic regression analysis was used to calculate the estimated propensity score for survival at 30 days for each patient. Patient characteristics including age, sex, comorbidities, initial hemodynamic variables, laboratory results considered to be associated with disease severity or mortality such as white blood cell count, hemoglobin levels, platelet levels, blood urea nitrogen levels, creatinine levels, albumin levels, and C-reactive protein levels were included in the multivariable analysis. A propensity score analysis of 1:1 matching was performed without replacement using the nearest neighbor method with a maximum caliper of 0.4 to generate matched pairs of patients. The standardized difference was calculated to compare group differences and assess the balance of the clinical characteristics of study subjects before and after propensity score matching. To verify the survival in the matched groups, the survival curves of the two groups according to whether empirical antibiotics were administered consistent with the guidelines were plotted using the Kaplan–Meier method, and the log-rank test was performed.

All data processing and statistical analyses were performed using Stata version 15.1 (StataCorp, College Station, TX, USA). A two-tailed *p* value of less than 0.05 was considered statistically significant.

## Results

### Clinical characteristics of the study population

**Fig. 1:**
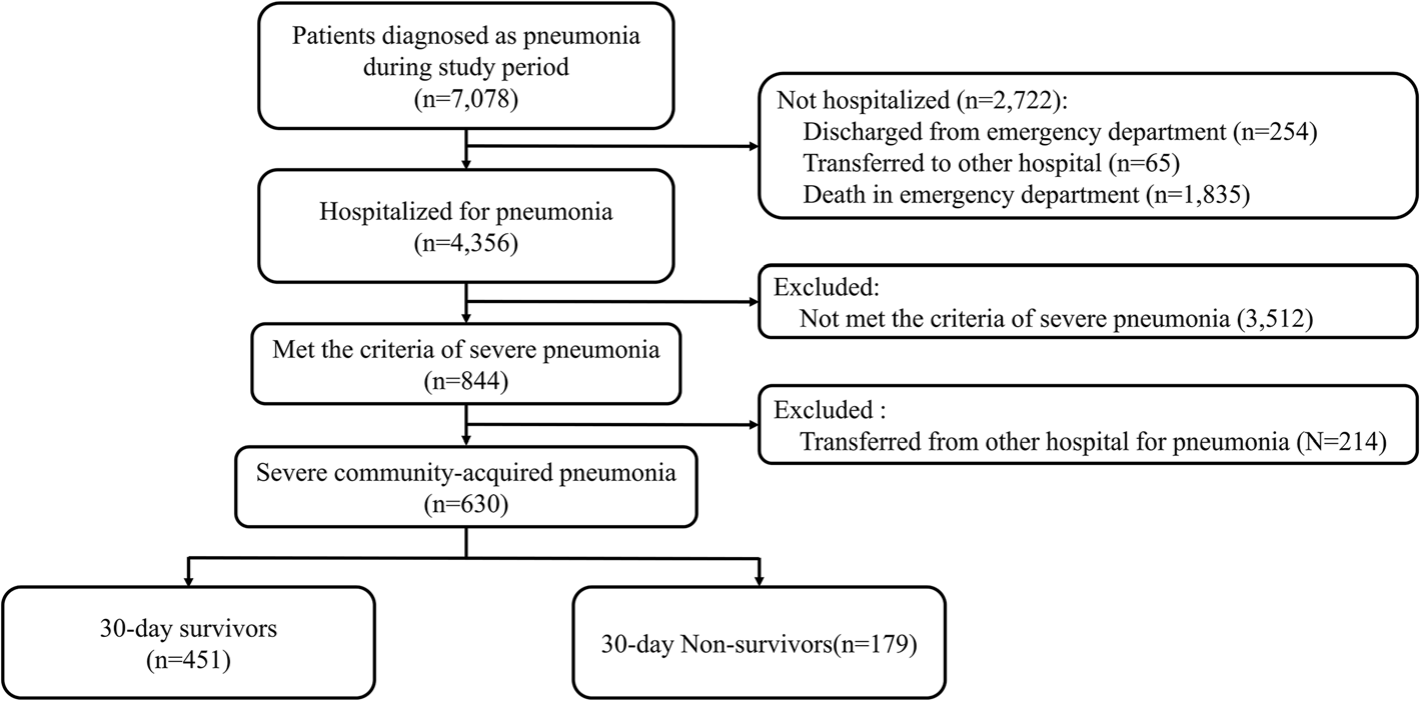
Flow chart of the study population

During the study period, 7078 patients were diagnosed with and underwent treatment for pneumonia in the ED. Of these patients, 2416 were discharged directly from the ED, 230 were transferred to other hospitals, and 76 died in the ED. In total, 4356 patients were hospitalized in the ED. Of these patients, 214 were transferred from another hospital and had already received antibiotics before visiting the ED, and 3512 patients did not meet the criteria for severe CAP. Thus, 630 patients were finally included in the analysis (Fig. 1). The 30-day mortality rate of the included patients was 28.4%.

Table 1 shows the baseline characteristics, initial hemodynamic variables, and laboratory findings of the enrolled patients according to whether empirical antibiotics were prescribed concordant with guidelines before and after propensity score matching. Before propensity score matching, the group prescribed guideline-concordant antibiotics was older, had higher frequencies of chronic obstructive lung disease, and had lower pneumonia severity index score than the group prescribed guideline-discordant antibiotics. The frequency of patients with multidrug-resistant pathogens isolated from sputum or blood cultures was higher in the group prescribed guideline-discordant antibiotics. Multidrug-resistant pathogens included *Pseudomonas aeruginosa*, MRSA, and extended-spectrum β-lactamase-producing gram-negative organisms. After propensity score matching, there were 255 patients in the group prescribed guideline-concordant antibiotics and the group prescribed guideline-discordant antibiotics, respectively. After matching, there was no difference in the clinical characteristics between the two groups (Table 1).

**Table 1:** Baseline characteristics according to the antibiotics prescribed before and after propensity score matching

	Before propensity score matching			After propensity score matching		
	Guideline-concordant antibiotics (*N* = 359)	Guideline-discordant antibiotics (*N* = 271)	SD	Guideline-concordant antibiotics (*N* = 255)	Guideline-discordant antibiotics (*N* = 255)	SD
Age	74.63 (11.56)	76.85 (11.66)	0.191	75.75 (11.16)	76.81 (11.86)	0.092
Sex (Male)	245 (68.2)	179 (66.1)	0.047	170 (66.7)	167 (65.5)	0.025
Comorbidities						
Diabetes mellitus	114 (31.8)	83 (30.6)	0.024	83 (32.5)	80 (31.4)	0.025
Hypertension	190 (52.9)	132 (48.7)	0.084	129 (50.6)	125 (49.0)	0.031
Chronic obstructive lung disease	43 (12.0)	23 (8.5)	0.115	24 (9.4)	23 (9.0)	0.014
Congestive heart failure	12 (3.3)	6 (2.2)	0.068	8 (3.1)	5 (2.0)	0.075
Chronic kidney disease	43 (12.0)	35 (12.9)	0.028	34 (13.3)	34 (13.3)	< 0.001
Chronic liver disease	30 (8.4)	13 (4.8)	0.144	15 (5.9)	13 (5.1)	0.034
Neoplastic diseases	109 (30.4)	69 (25.6)	0.107	67 (26.3)	67 (26.3)	< 0.001
Cerebrovascular disease	104 (29.0)	101 (37.3)	0.177	84 (32.9)	91 (35.7)	0.058
Initial hemodynamic variables						
Mean arterial pressure (mmHg)	88.51 (21.97)	87.89 (21.03)	0.029	87.53 (21.72)	87.76 (21.28)	0.011
Heart rate (beat/min)	107.58 (25.38)	104.27 (25.47)	0.130	104.84 (25.10)	104.76 (25.74)	0.003
Respiratory rate (breath/min)	24.89 (7.01)	25.17 (7.08)	0.040	24.82 (7.03)	25.15 (7.02)	0.046
Body temperature (°C)	37.39 (1.16)	37.15 (1.11)	0.213	37.23 (1.15)	37.19 (1.10)	0.034
Laboratory results						
White blood cell (10^3^/μL)	11.26 (7.71)	12.59 (7.71)	0.172	12.14 (8.01)	12.40 (7.58)	0.033
Hemoglobin (g/dL)	11.56 (2.31)	11.39 (2.29)	0.074	11.38 (2.41)	11.45 (2.31)	0.026
Platelet (10^3^/μL)	211.66 (110.39)	218.59 (117.09)	0.061	214.47 (110.81)	216.12 (116.32)	0.015
BUN (mg/dL)	32.83 (22.78)	38.66 (26.56)	0.236	35.98 (24.99)	38.00 (25.92)	0.079
Creatinine (mg/dL)	1.58 (1.59)	1.69 (1.65)	0.069	1.69 (1.80)	1.69 (1.68)	0.003
Albumin (g/dL)	3.08 (0.55)	3.00 (0.63)	0.138	3.05 (0.55)	3.00 (0.63)	0.072
CRP (mg/dL)	14.00 (9.11)	12.58 (9.04)	0.157	12.77 (8.68)	12.69 (9.10)	0.008
PSI score	132.19 (34.63)	140.03 (38.26)	0.215	136.24 (35.43)	139.08 (38.75)	0.076
Multidrug-resistant pathogens isolated^a^	32 (8.9)	42 (15.5)	0.202	30 (11.8)	36 (14.1)	0.070

Data are expressed as mean (standard deviation) or n (%) as appropriate. *BUN* blood urea nitrogen, *CRP* C-reactive protein, *PSI* pneumonia severity index

^a^Multidrug-resistant pathogens includes *Pseudomonas aeruginosa*, Methicillin-Resistant *Staphylococcus aureus*, and ESBL-producing Gram-negative organisms

The standardized difference was calculated to compare variables before and after the propensity score matching. Imbalance was defined as the standardized difference greater than 0.10

Table 2 shows the prescribed initial antibiotics in the ED according to 30-day mortality. Overall, the antibiotic prescription rate consistent with the guidelines was 57.0% (359/630). The proportion of antibiotic prescriptions consistent with the guidelines was significantly higher in survivors than in non-survivors (60.8% vs. 47.5%, respectively, *p* = 0.002). Among the combination of initial empirical antibiotics prescribed to patients consistent with the guidelines, the most common combination was antipseudomonal β-lactam + fluoroquinolone (47.1%), followed by non-antipseudomonal β-lactam + azithromycin (30.1%). Regarding antibiotics prescribed that were not consistent with the guidelines, a β-lactam alone (58.3%), a β-lactam + anti-anaerobes (31.0%), and a fluoroquinolone alone (7.0%) were often prescribed.

**Table 2:** Initial antibiotics treatment and 30-day mortality of the patients

	Total (*n* = 630)	Survivors (*n* = 451)	Non-survivors (*n* = 179)
Guideline concordant group	359	274 (60.8)	85 (47.5)
Antipseudomonal β-lactam + fluoroquinolone	169 (47.1)	120 (43.8)	49 (57.6)
Non-antipseudomonal β-lactam + azithromycin	108 (30.1)	92 (33.6)	16 (18.8)
Non-antipseudomonal β-lactam + fluoroquinolone	82 (22.8)	62 (22.6)	20 (23.5)
Guideline discordant group	271	177 (39.3)	94 (52.5)
β-lactam	158 (58.3)	96 (54.2)	62 (66.0)
β-lactam + anti-anaerobes	84 (31.0)	56 (31.6)	28 (29.8)
β-lactam + vancomycin	1 (0.3)	1 (0.6)	0 (0.0)
Fluoroquinolone	19 (7.0)	16 (9.0)	3 (3.2)
Carbapenem	9 (3.3)	8 (4.5)	1 (1.0)

Data are expressed as n (%)

Table 3 shows the pathogens that were isolated from sputum or blood cultures. The most commonly isolated pathogen was *Klebsiella pneumoniae*, followed by *Staphylococcus aureus* and *Pseudomonas aeruginosa*.

**Table 3:** Pathogens isolated from blood or sputum cultures

	Total	Guideline-concordant antibiotics	Guideline-discordant antibiotics
*Streptococcus pneumoniae*	18 (7.5%)	15 (11.9%)	3 (2.6%)
*Group B streptococcus*	8 (3.3%)	4 (3.2%)	4 (3.5%)
*Escherichia coli*	23 (9.5%)	13 (10.3%)	10 (8.7%)
*Pseudomonas aeruginosa*	27 (11.2%)	10 (7.9%)	17 (14.8%)
*Staphylococcus aureus*	50 (20.7%)	24 (19.0%)	26 (22.6%)
*Acinetobacter baumanii*	6 (2.5%)	2 (1.6%)	4 (3.5%)
*Haemophilus influenzae*	5 (2.1%)	4 (3.2%)	1 (0.9%)
*Serratia marcescens*	8 (3.3%)	2 (1.6%)	6 (5.2%)
*Stenotrophonas maltophillia*	2 (0.8%)	1 (0.8%)	1 (0.9%)
*Group G streptococcus*	2 (0.8%)	1 (0.8%)	1 (0.9%)
*Group A streptococcus*	2 (0.8%)	1 (0.8%)	1 (0.9%)
*Klebsiella pneumoniae*	67 (27.8%)	37 (29.4%)	30 (26.1%)
*Klebsiella oxytoca*	8 (3.3%)	3 (2.4%)	5 (4.3%)
*Providencia stuartii*	3 (1.2%)	2 (1.6%)	1 (0.9%)
*Enterobacter cloacae*	7 (2.9%)	4 (3.2%)	3 (2.6%)
*Enterobacter aerogenes*	1 (0.4%)	1 (0.8%)	0 (0.0%)
*Proteus mirabilis*	4 (1.7%)	2 (1.6%)	2 (1.7%)

### Antibiotic prescription consistent with the guidelines and 30-day mortality

**Fig. 2:**
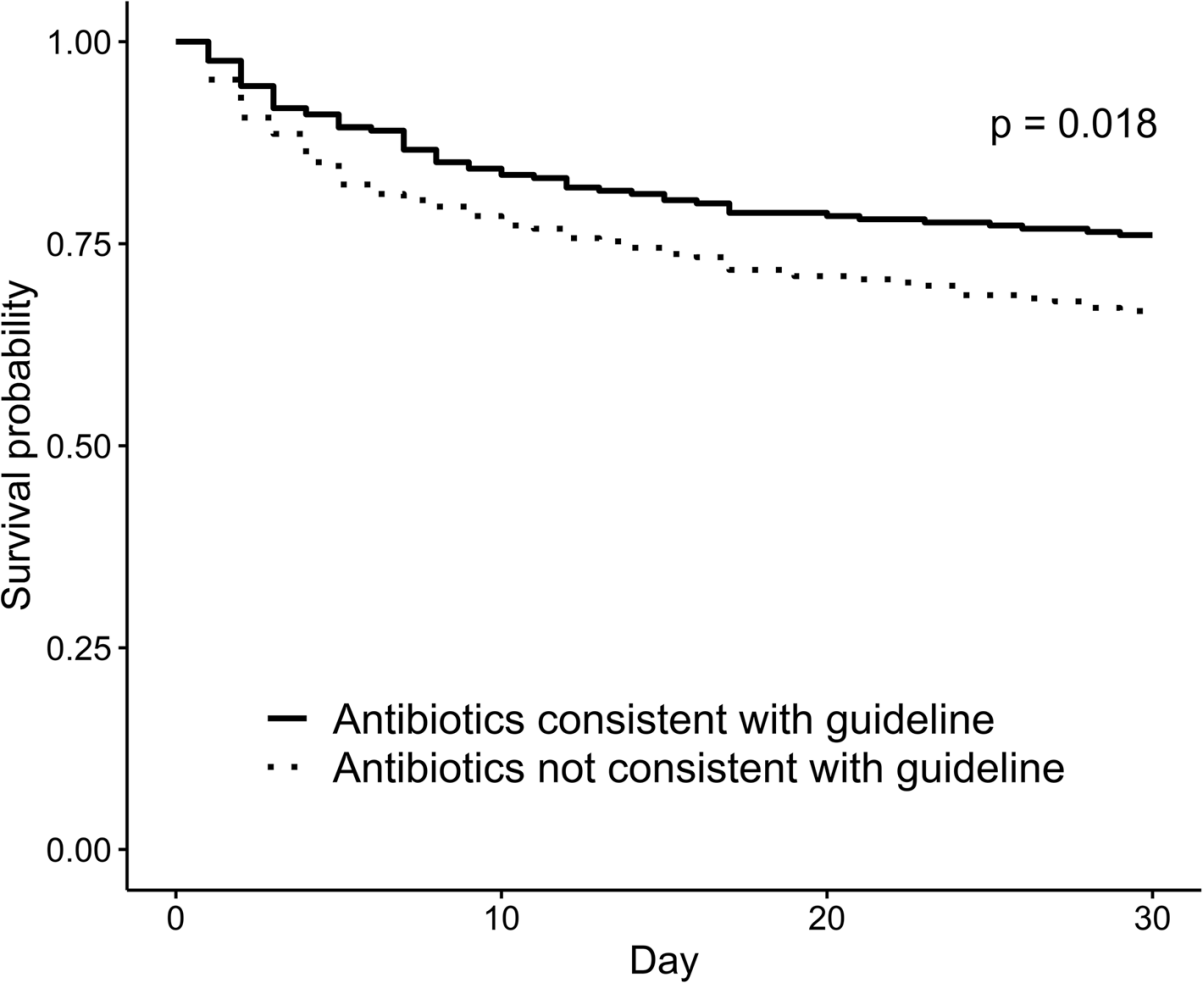
Kaplan-Meier curve of the two groups divided by whether initial empirical antibiotics were administered concordant to the Infectious Diseases Society of America/American Thoracic Society guidelines

Before propensity score matching, the 30-day mortality rates of the group prescribed guideline-concordant antibiotics and the group prescribed guideline-discordant antibiotics were 23.7% (85/359) and 34.7% (94/271), respectively (*p* = 0.003). In multivariable Cox proportional hazards regression analysis, summarized in Table 4, the prescription of guideline-discordant antibiotics was significantly associated with the 30-day mortality (hazard ratio 1.43, 95% CI 1.05–1.93, *p* = 0.022). In the analysis after propensity score matching, the 30-day mortality was also lower in the group prescribed guideline-concordant antibiotics than in the group prescribed guideline-discordant antibiotics (23.9% vs. 33.3%, *p* = 0.024). Kaplan–Meier survival analysis with the log-rank test, performed in patients after propensity score matching, revealed that antibiotic prescriptions consistent with the guidelines resulted in higher survival rates at 30 days (Fig. 2, *p* = 0.018).

**Table 4:** Multivariable Cox regression analysis for 30-day mortality

	Hazard ratio	95% CI	p
Age	1.01	0.99–1.02	0.524
Sex (Male)	0.94	0.67–1.32	0.727
Chronic obstructive lung disease	0.71	0.41–1.23	0.217
Chronic liver disease	1.16	0.65–2.06	0.621
Neoplastic diseases	1.49	1.05–2.13	0.027
Cerebrovascular disease	1.02	0.73–1.41	0.929
Heart rate	1.00	1.00–1.01	0.326
Heart rate	0.84	0.72–0.98	0.027
White blood cell	0.99	0.97–1.01	0.227
BUN	1.00	0.99–1.01	0.878
Albumin	0.54	0.40–0.72	< 0.001
CRP	1.01	0.99–1.03	0.361
PSI score	1.01	1.00–1.02	0.001
Multidrug-resistant pathogens isolated	1.43	0.96–2.14	0.078
Guideline-discordant antibiotics	1.43	1.05–1.93	0.022

*CI* confidence interval, *BUN* blood urea nitrogen, *CRP* C-reactive protein, *PSI* pneumonia severity index

## Discussion

In the present study, among ED patients with severe CAP, we found that a high proportion of ED patients (43.0%) with severe CAP were prescribed antibiotics discordant with the IDSA/ATS guidelines. In addition, the results of this study revealed that patients who received initial empirical antibiotics concordant with the guidelines had lower 30-day mortality than those who received antibiotics discordant with the guidelines before and after propensity score matching.

In a recent prospective observational cohort study among hospitalized patients with CAP, the prescription of antibiotics consistent with the guidelines was associated with short hospital length of stay and improved survival [18]. Several previous studies that included a subgroup of patients with severe CAP also reported that prescription of antibiotics consistent with the guidelines was associated with good prognosis. Menéndez et al. [12] investigated the relationship between the selection and timing of administration of antibiotics in patients with CAP and patient mortality, and in patients with CAP with severe sepsis (as per the Second International Sepsis Definition Consensus), the administration of antibiotics concordant with the guidelines within 6 h could improve the outcomes of the patients. Wongsurakiat et al. [14] investigated the association between guideline-concordant prescription of antibiotics and in-hospital mortality in patients who met the criteria for severe CAP and were admitted to the general ward and reported that the group received guideline-discordant antibiotics had worse outcomes. Other previous studies that included patients with CAP admitted to the ICU or patients with CAP who required mechanical ventilation also showed that guideline-concordant prescription of antibiotics was associated with good patient outcomes [11, 13]. In the present study, which was conducted in patients with severe CAP who were hospitalized in the ED, the administration of initial empirical antibiotics consistent with the guidelines in the ED was independently associated with improved patient outcomes.

Clinical practice guidelines are designed to improve the quality of care by assisting in clinical decision-making through an evidence-based approach and the use of standardized treatments. To date, several guidelines related to the treatment of CAP have been published [5, 9, 10, 17], and some studies have shown that providing treatments according to these guidelines is helpful for improving the prognosis of patients [11–14, 18]. However, from the results of previous studies, real-world practices do not strictly follow these guidelines. In the present study, the prescription of initial empirical antibiotics not consistent with the guidelines reached 43% of all patients. In the treatment of patients with severe CAP, insufficient antimicrobial coverage of initial empirical antibiotics may be one of the major causes of poor patient outcomes. Although the recommended antibiotics may vary depending on the regional antibiotic susceptibility pattern, most of the guidelines for the treatment of CAP recommend the administration of a combination of antibiotics in patients with high disease severity to cover a sufficient antimicrobial range. However, as per the results of our study, β-lactams or fluoroquinolones were administered alone in 28.1% of all patients. In these patients, insufficient antimicrobial coverage is estimated to be one of the major causes of poor outcomes.

This study has several limitations. First, this study was a retrospective analysis of data collected at a single institution; hence, it would be difficult to directly generalize these results to other institutions. Therefore, further studies are required to determine the generalizability of the findings. Second, changes in antibiotics depending on the hospital course of the patients during the ED stay or during hospitalization after the administration of initial empirical antibiotics were not investigated. However, escalation or de-escalation of antibiotic regimens is usually performed after obtaining culture results or when the patient’s clinical condition changes. Moreover, the results of our study highlight the importance of prescribing initial empirical antibiotics concordant with the guidelines in the ED. Third, several previous studies have reported that not only the selection of initial empirical antibiotics but also the time to the first administration of antibiotics affects patient outcomes [12, 19]; however, the timing of antibiotic administration was not included in the analysis.

## Conclusions

The prevalence of antibiotic prescriptions consistent with the guidelines for severe CAP seemed to be low in the ED, and this variable was independently associated with 30-day survival.

## Data Availability

The datasets used and/or analysed during the current study are available from the corresponding author on reasonable request.

## Abbreviations

CAP: Community-acquired pneumonia
MRSA: Methicillin-resistant *Staphylococcus aureus*
ICU: Intensive care unit
ED: Emergency department
IDSA-ATS: Infectious Diseases Society of America/American Thoracic Society
PSI: Pneumonia severity index
